# Simulated heat waves reduce cognitive and motor performance of an endotherm

**DOI:** 10.1002/ece3.7194

**Published:** 2021-01-26

**Authors:** Raymond M. Danner, Casey M. Coomes, Elizabeth P. Derryberry

**Affiliations:** ^1^ Department of Biology and Marine Biology University of North Carolina Wilmington Wilmington NC USA; ^2^ Department of Ecology and Evolutionary Biology Tulane University New Orleans LA USA; ^3^ Department of Ecology and Evolutionary Biology University of Tennessee Knoxville TN USA

**Keywords:** behavior, climate change, cognition, cognitive performance, heat wave, zebra finch

## Abstract

1. Heat waves cause mass mortality of animals, including humans, across the globe annually, which has drawn new attention to how animals cope with high air temperatures. Recent field research has explored behavioral responses to high air temperatures, which can influence reproductive success and mortality.
2. Less well studied are the effects of high air temperatures on cognition, which may underlie behavioral changes. Specifically, it is poorly known if cognitive declines occur at high temperatures, and if cognitive and motor components of behavior are similarly affected.
3. We tested how well zebra finches (*Taeniopygia guttata castanotis*), a model for cognition research, performed two learned foraging tasks (color association and detour‐reaching) at mild (22°C) and high (43 and 44°C) air temperatures that occur naturally in their range. We habituated birds to the trial conditions and temperatures on days preceding the test trials and at the trial temperature for 30 min immediately prior to each test trial. Trials lasted less than 10 min. At high air temperatures, zebra finches exhibited heat dissipation behaviors during most tasks, suggesting thermoregulatory challenge.
4. Cognitive performance declined at high air temperatures in two of three measures: Color association was unaffected, but birds missed more food rewards, and did more unproductive behaviors. Motor performance declined at high temperatures on the color association task, including longer times to complete the task, move between food rewards, and process individual seeds. Performance declines varied among components of behavior and among individuals.
5. We combined our behavioral data with existing climate data and predicted that in the austral summer of 2018–2019, zebra finches experienced air temperatures that caused cognitive and motor declines in our captive birds in 34% and 45% of their Australian range, respectively.
6. This study provides novel experimental evidence that high air temperatures cause cognitive and motor performance decline in birds. Further, our results provide insights to how those declines might affect bird ecology and evolution. First, differences in declines among behavioral components may allow identification of behaviors that are most susceptible to decline in the wild. Second, variation in performance declines and heat dissipation behaviors among individuals suggests variability in heat tolerance, which could lead to differential fitness in the wild. Last, these results suggest that high air temperatures cause cognitive declines in the wild and that understanding cognition could help refine predictive models of population persistence.

## Graphical abstract

To understand how high temperatures affect cognitive and motor performance, we taught zebra finches to perform tasks and then asked whether their performance declined at high temperatures. At high temperatures, zebra finches showed significant declines in some, but not all, cognitive tasks and exhibited slower movements. We then performed a mapping study and found that zebra finches experience temperatures in the wild that caused cognitive and muscle decline in our experiment.

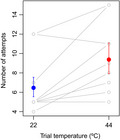

## 1 INTRODUCTION

Life on Earth is currently experiencing the hottest air temperatures in recent history with continued increases in temperature across large areas of land and ocean (Wallace‐Wells, 2020). The last 5 years have been the hottest in 140 years of recorded history (NOAA, 2019a), with July of 2019 being the hottest month ever recorded (NOAA, 2019b). Not only are average air temperatures higher, but there is also an increase in the frequency of “extreme events,” including increases in extreme high temperatures (Drumond et al., 2020; Jentsch et al., 2007). Whereas these hot extremes were limited to less than 1% of the Earth's surface in the past, extreme anomalies in high air temperatures now cover more than 10% of land area (Hansen et al., 2012). Thus, terrestrial organisms are experiencing not only a long‐term increase in air temperature but also marked stretches of high heat, or “heat waves.” Despite this global challenge, we know relatively little about the impact of heat waves on animal populations (Jentsch et al., 2007; Stillman, 2019).

Predicting how animals will respond to high air temperatures requires knowledge of its impact on animal behavior and physiology, as well as the genetic capacity of a species to adapt to changing temperatures (Huey et al., 2012). Our knowledge of these effects is woefully limited for most species, inhibiting our ability to make specific predictions about the effects of climate change or recurring heat waves on populations. In particular, we know little about how high air temperatures affect the behavior and physiology of endotherms, such as humans, mammals, birds, and many fish, in comparison to the literature on effects of heat on performance in ectotherms, such as reptiles, amphibians, and insects (reviewed in Angilletta, 2009). Filling this knowledge gap is particularly pressing given mass mortality events among birds (McKechnie & Wolf, 2010), small mammals (Ratnayake et al., 2019), and humans (Zhao et al., 2018) during heat waves, and has spurred recent interest in measuring the effects of elevated, sublethal air temperatures in endotherms (Hurley et al., 2018; Jimenez & Williams, 2014; Lovegrove et al., 2013; McKechnie, Smit, et al., 2016; McKechnie, Whitfield, et al., 2016; du Plessis et al., 2012).

Emerging research suggests that heat affects fitness‐related behaviors, such as foraging or breeding, and that these sublethal effects occur at air temperatures well below those that cause death (Cunningham et al., 2013). For example, recent field studies provide evidence that birds forage less (Carroll et al., 2015; Funghi et al., 2019; Goldstein, 1984) and less efficiently (du Plessis et al., 2012; van de Ven et al., 2019), spend less time incubating eggs and provisioning young (Cunningham et al., 2013, 2015; Wiley & Ridley, 2016), and sing less in hot weather (Luther & Danner, 2016). Similarly, high air temperatures reduce feed intake in livestock, including poultry (Nawab et al., 2018), and reduced time spent foraging in a free‐living montane mammal (Hall & Chalfoun, 2019). Because performance of foraging and mating behaviors can affect survival and reproductive success (Borgia, 1985; Sonnenberg et al., 2019), these sublethal behavioral effects of heat may be essential to predicting species' persistence in the face of high and rising temperatures (Conradie et al., 2019, 2020; McKechnie et al., 2012; Sinervo et al., 2010).

Studies that experimentally evaluate behavioral effects of high air temperatures in birds are rare. Controlled experimental studies are valuable, allowing for behavioral assays that are not logistically possible in the wild and reducing the number of potential explanatory variables, which can help identify mechanisms. Past experimental, physiological studies include notes that birds lose the ability of coordinated movement when at or nearing lethal air temperatures (Dawson, 1954; Whitfield et al., 2015), which could affect behavioral performance. Recently, Coomes et al. (2019) found that high air temperatures reduced female birds’ ability to discriminate between conspecific and heterospecific sexual signals. Results from these few experimental studies highlight the need to investigate the effect of high temperatures on bird behavior.

High air temperatures and resulting hyperthermia could limit both motor and cognitive performance components of behaviors. Motor performance (e.g., speed) may decline when individuals slow movements in high temperatures (e.g., in humans and rats: Fuller et al., 1998; Morrison et al., 2004; Racinais et al., 2008) as animals seek to reduce heat generation (hypothesized by Tucker et al., 2004) or experience declines in muscle performance (Racinais et al., 2008). However, it is not known whether or how reductions in motor speed would affect performance of foraging or mating behaviors. Hyperthermia may also affect cognitive function. Studies on humans have shown that high air temperatures reduce performance on tests of memory, but not attention (Racinais et al., 2008), as well as reduced performance on work tasks (Mazloumi et al., 2014). In other animals, a recent experimental study demonstrates female birds become less discriminating among male signals when exposed to high air temperatures during an operant conditioning assay of preference (Coomes et al., 2019). Together, these studies indicate the need to consider both motor and cognitive components of behavior when measuring the effects of high air temperature on performance.

**FIGURE 1:**
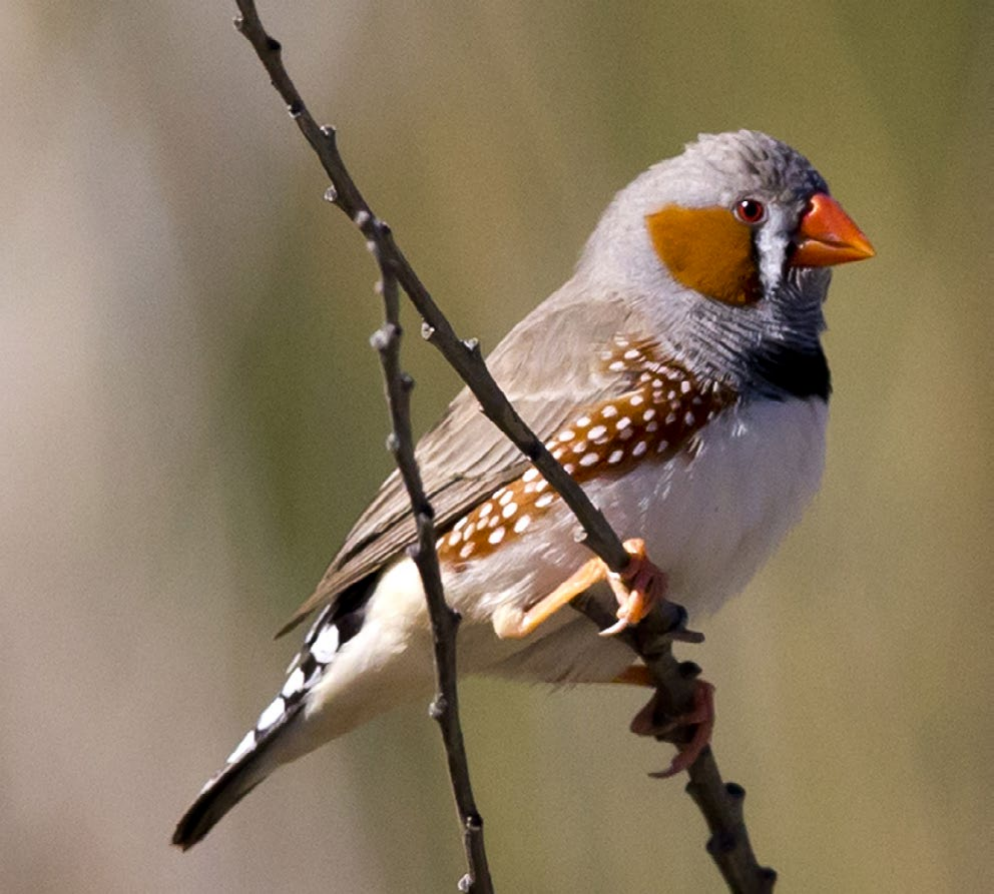
Photo of a male zebra finch (*Taeniopygia guttata*). Photo by Jim Bendon

Here, we test the effect of simulated heat waves on cognitive and motor performance of foraging assays, using zebra finches (Figure 1), *Taeniopygia guttata*, an emerging model system for studies on cognition (Healy et al., 2010). Zebra finches live in the hot, arid interior of Australia, where they experience heat waves (Funghi et al., 2019). Cognitive tasks measure how animals collect, retain, and use information from the environment, which is essential in fitness‐related behaviors, including foraging, predator avoidance, and mating (Morand‐Ferron et al., 2016). We tested zebra finches using two different established assays of cognitive performance: a color association task and a detour‐reaching task (Boogert et al., 2011). Color association tasks measure associative learning, which has been linked to foraging success in free‐living animals (Cole et al., 2012; Raine & Chittka, 2008). Detour‐reaching tasks measure self‐control, also referred to as inhibitory control, which is important for making decisions related to foraging (MacLean et al., 2014). For both tasks, we tested performance at typical housing air temperatures and at temperatures above the published thermoneutral zone for zebra finches. The zebra finch's thermoneutral zone is approximately 36–42°C and mortality is expected above 45°C (Bech et al., 2004; Cade et al., 1965; Calder, 1964; Rønning et al., 2005). There is no apparent difference between domesticated and wild zebra finches in their physiological response to high air temperatures (Calder, 1964). We predicted that high temperatures would cause zebra finches to perform heat dissipation behaviors, which would suggest thermoregulatory challenges, and experience reduced cognitive and motor performance on both tasks. To place our results in a relevant ecological context, we then built maps from climate data to determine whether wild zebra finches in their native range in Australia would experience air temperatures at which captive birds exhibited changes in behavioral performance. Together, our work represents a novel experimental test of the sublethal effects of high air temperatures on cognitive and motor performance in an endotherm and further places our results in the context of the organism's environment.

## 2 MATERIALS AND METHODS

### 2.1 Subjects

We obtained two groups (*n* = 12 and 12, respectively) of adult male zebra finches from an avian breeder (Magnolia Farms). Like the majority of breeding facilities, this breeder provides individuals from the subspecies *T. g*. *castanotis*. One group was housed at the senior author's institution for the color association task, and one group was housed at the lead author's institution for the detour‐reaching task. We housed birds individually for trials in wire cages with perches and cuttlebone and provided food and water ad libitum. We kept birds on a 12:12 light–dark cycle at air temperature of 22°C and relative humidity of 50%–75%.

### 2.2 Heat dissipation behaviors

We tested if birds showed heat dissipation behaviors during behavioral tasks at four air temperatures (22, 40, 43, and 44°C). Our experimental temperatures spanned a range of published effects on zebra finch body temperatures. Calder (1964) found that zebra finches experience mild hyperthermia above 30°C, and by 44°C had increased body (i.e., cloacal) temperature by 3.5°C. All data and scripts are available on Zenodo (https://zenodo.org/record/4437461).

### 2.3 Color association task

We taught birds to remove lids fitted snugly into six wells drilled in a gray composite plastic block (10 × 14 cm) in order to reach millet seed following Boogert et al. (2011). This task had three phases: habitation, training, and testing. Habitation and training included five stages of trials: (a) no lids, (b) lids next to wells, (c) lids tipped into wells, and (d) lids covering wells. Birds had to eat from at least two of four baited wells within two minutes to pass a trial and had to pass three out of four consecutive trials to move to the next stage. We baited wells randomly and changed every trial. For the last stage, (e) birds were taught to associate seed with only one randomly assigned color of lid (yellow or blue). For this stage, we used two blocks and baited the six wells with either yellow or blue lids. To pass a trial, birds had to flip the four lids of the reward color before trying lids of the other color. To pass the stage, birds had to pass six out of seven consecutive trials. Birds stuck on a stage returned to the previous stage. Birds that did not complete a stage in 2 weeks (60 trials) were removed. To ensure motivation, we removed food from cages five hours before trials began. After trial completion, we performed a motivation test placing food dishes in cages and recording the time it took birds to approach. All birds approached food dishes in less than one minute, which is considered sufficiently motivated (Boogert et al., 2011). We removed water immediately before the trial began.

We included only birds that learned the color association task in test trials (*n* = 6, 50% of original group). We then tested these birds with two blocks with their reward color baited. We repeated the color association task for each bird at three air temperatures with treatment order randomized within temperature pairs: 22 and 40°C, and then 22 and 43°C. We maintained relative humidity at approximately 65% in trials at 22°C because this was similar to the housing conditions. For the high‐temperature trials, we maintained relative humidity at 25% to ensure that it did not constrain evaporative cooling (van Dyk et al., 2019; Gerson et al., 2014). Birds underwent trials in home cages placed in an environmental chamber (Conviron A1000). We recorded all test trials using webcams mounted inside the environmental chamber. We took six behavioral measures from these trials: latency to begin the task (time from trays were added to the first lid flipped), time to complete the task (from first to last lids flipped), time to process individual seeds (from picking up a seed to the husk falling from the mouth), whether or not the bird missed food rewards (i.e., ate less than all three seeds in each well before moving to the next well), the proportion of lids that birds flipped correctly, and whether or not the bird panted during the trial. To test the effect of temperature on the first five behaviors, we built generalized linear mixed models with functions “lme” (Pinheiro et al., 2020) and “glmer” (Bates et al., 2015) in R (R Core Team, 2020) following Zuur et al. (2009). Models included a factor variable for trial temperature, and all models included bird identification as a random effect in order to account for repeated measures within individuals. For models with continuous data, we use a Gaussian error distribution, and for models with binary or proportional data, we used a binomial error distribution. We present effect sizes (*B* or *η*) ± standard errors estimated from the models, measures of support for models, including test statistics (*t*, *F*, or *z*), *p*‐values for specific terms, test statistics, and *p*‐values from likelihood‐ratio tests comparing models with and without air temperature, and *r* ^2^ for full models when available using package r2glmm (Jaeger, 2017). To describe interindividual variation, we present the standard deviation of interbird variation from the linear models. Models with binomial error distributions had dispersion parameters of 1.5 and 2.0, respectively, following Bolker (2020). All R scripts and data are available in Zenodo (https://zenodo.org/record/4437461).

### 2.4 Detour‐reaching task

Birds were taught to reach into a tube to retrieve a food reward following Boogert et al. (2011). This task had three phases: habituation, training, and testing. For all phases, a trial consisted of placing a tube (5 cm length, 4 cm diameter) mounted on a thin piece of wood in a bird's home cage for 10 min with a food reward (freshly killed mini‐mealworm) at the center of the tube. Trials were repeated sequentially until the bird passed each phase, with no more than 20 trials per day. During the habituation phase, birds were taught to associate an opaque tube with food and passed habituation by taking food in three consecutive trials. Birds were in the main housing room during habituation but visually isolated from other birds with opaque black plastic partitions. For training and testing, we moved birds in their home cage to an environmental chamber (Caron 7000‐10). During the training phase, we exposed birds to the same air temperature and relative humidity as the main housing room (22°C, 55%–65% rH). Birds were then taught to reach around the opaque tube to remove food instead of pecking on the surface. An individual passed training when it no longer pecked on the side of the tube before removing the food in four out of five consecutive trials.

We included only birds that passed the training phase in the testing phase (*n* = 9, 75% of original group). In the testing phase, birds were presented with baited clear tubes and passed when they no longer pecked on the side of the tube before removing the food in four out of five consecutive trials. We repeated the testing phase at two air temperatures: 22 and 44°C in a repeated measures design with treatment order randomized across birds. We maintained relative humidity at the same levels as in the color association task. We recorded all test trials to a computer using webcams. Closely matching our protocol for the color association task, we removed food from cages four hours before trials began to ensure motivation and removed water from cages immediately before the trial began.

We tested for differences in the number of trials to pass the task between temperature treatments by fitting generalized linear mixed models using function “glmer” (Bates et al., 2015) that included trial temperature as a fixed factor variable and bird identification as a random effect. In a separate model, we tested if trial number influenced performance. To describe interindividual variation, we present the standard deviation of interbird variation from the linear models. For both models, we used Poisson error distributions; models were not overdispersed (dispersion parameters 0.53 and 0.85, respectively, chi‐square test both *p* ≥ 0.62, following Bolker, 2020). We calculated *r* ^2^ using package r2glmm (Jaeger, 2017) and plotted raw data with model predictions based on bootstrapping (Duursma, 2020).

### 2.5 Ethical treatment

The IACUC at each institution approved housing and experimental conditions. We habituated birds to thermal chambers immediately prior to the test trials and on days preceding test trials. Habituation immediately before the trial was for 30 min at trial air temperature. For the color association task, we also habituated birds to the environmental chamber on separate days at 22°C for 2 hr and at an elevated temperature (36°C) for 1.5 hr. For the detour‐reaching task, we also habituated the birds to the chamber on a separate day at 22°C for 1 hr. We monitored birds continuously during trials via webcams and removed one bird from a test trial because it exhibited signs of prolonged stress. Following each trial, we supplied fresh food and water to the birds’ cages.

### 2.6 Air temperature in native range

We mapped air temperature in the native range of the zebra finch subspecies used in our captive studies (*T. g*. *castanotis*). This subspecies is found across mainland Australia, generally in the more arid areas. The other, nominate subspecies of zebra finch is found in the Lesser Sunda Islands and coastal areas of Australia (BirdLife International, 2016; Sullivan et al., 2009). We downloaded daily maximum air temperature maps from the Australian Bureau of Meteorology (http://www.bom.gov.au/jsp/awap/temp/archive.jsp). These maps are interpolated based on data from several hundred weather stations around Australia. We used the bindings for the Geospatial Data Abstraction Library (Bivand et al., 2014) as well as the raster package (Hijmans & van Etten, 2020) in R (R Core Team, 2020) to read these maps and to calculate the number of days that reached air temperatures of 40°C and then 44°C for each geographical coordinate. We then overlayed the outline of Australia using the maps package (Brownrigg, 2021). Finally, we clipped the air temperature data to the zebra finch range. We obtained this range from BirdLife International (BirdLife International, 2016) and verified the range by ensuring that zebra finch sightings (eBird: Sullivan et al., 2009) and museum collection sites (VertNet: vertnet.org) were within that range.

## 3 RESULTS

### 3.1 Heat dissipation behaviors

**FIGURE 2:**
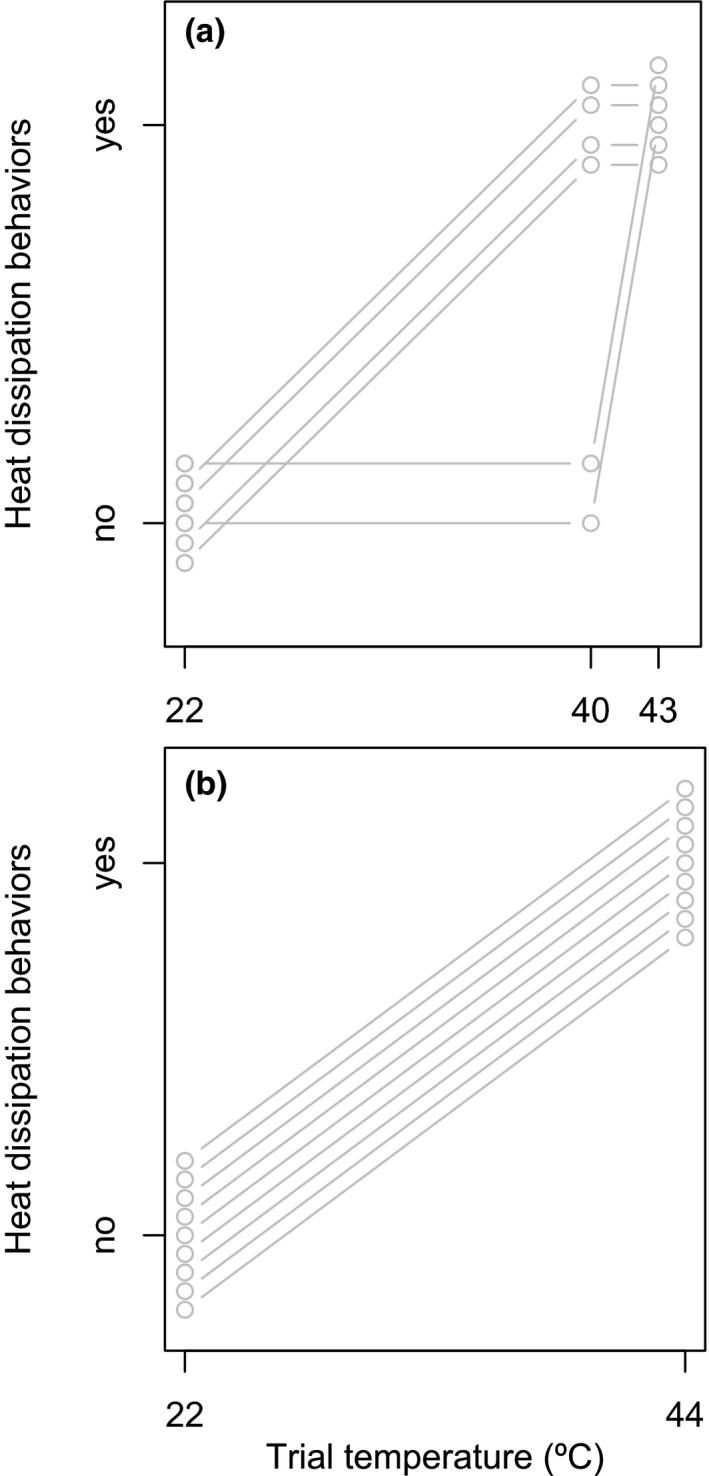
Zebra finches performed heat dissipation behaviors (panting, wing spreading, and taller posture) at air temperatures of 40°C and above on a color association task (a) and a detour‐reaching task (b). Gray lines connect repeated measurements for individuals (*n* = 6 and 9, respectively). Data points offset vertically to show all data

When exposed to higher air temperatures, birds showed heat dissipation behaviors, including panting, wing spreading, and taller posture (Figure 2). At low temperatures (22°C), birds never showed these behaviors (*n* = 9 in detour‐reaching study, *n* = 6 in color association study). At 40°C, four of six birds showed all three behaviors during color association trials (note that detour‐reaching trials were not conducted at this temperature). At 43 and 44°C, all birds (*n* = 15) showed all three thermoregulatory behaviors during trials for both color association and detour‐reaching tasks. These data suggest variability in heat tolerance.

### 3.2 Color association task

**FIGURE 3:**
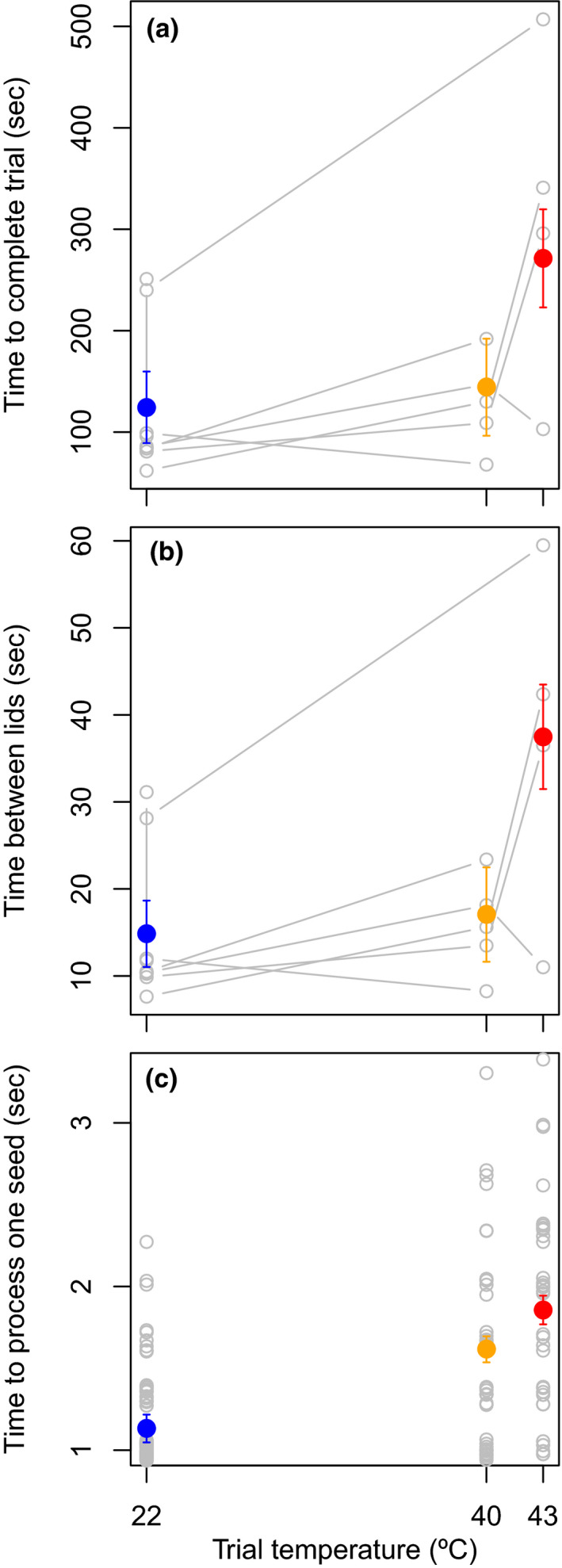
Zebra finches required longer to complete a color association task at higher air temperatures. This was reflected in total time to complete the trial (a) and resulted from longer times between flipping correct lids (b) and slower seed processing speeds (c) (*p* < 0.05 for all measures at 43°C and *p* < 0.05 for time to process a seed at 40°C). Filled dots and error bars represent model‐based predictions and standard errors; gray lines connect repeated measurements for individuals (*n* = 6). We did not provide lines in panel c because there were several measurements per individual at each temperature; instead, we added a small amount of random variation to values on the *y*‐axis to help visualize the points

Motor performance on the color association task declined at high air temperatures. Compared to the low air temperature (22°C), the time to finish the task was slightly longer per individual at 40°C, though not significant (Figure 3a,b = 20 s ± 49 s.e., *t*_12_ = 0.415, *p* < 0.69), and was significantly longer at 43°C (*B* = 147 s ± 48 s.e., *t*_12_ = 3.0, *p* < 0.011, likelihood‐ratio test: *p* < 0.015, *r* ^2^_adjusted_ = 0.47, sd of interbird variation = 49). One individual took four times longer to complete the trial at 43°C than at 22°C. The slower completion times at higher temperatures were attributed to at least two factors. First, birds paused to perform thermoregulatory behaviors, which caused them to take longer between flipping lids of the correct color (Figure 3b). The time between correct flips was slightly longer per individual at 40°C, though not significant (*B* = 2 s ± 5 s.e., *t*_11_ = 0.41, *p* < 0.7), and was significantly longer at 43°C (*B* = 23 s ± 6 s.e., *t*_11_ = 3.8, *p* < 0.004, likelihood‐ratio test: *p* < 0.03, *r* ^2^ = 0.60, sd of interbird variation = 4.8). Second, birds processed food rewards more slowly at higher temperatures (Figure 3c). Compared to 22°C, the time to eat a seed was significantly longer at both 40°C (*B* = 0.48 s ± 0.08 s.e., *t*_151_ = 6.1, *p* < 0.0001) and 43°C (*B* = 0.72 s ± 0.09 s.e., *t*_151_ = 8.2, *p* < 0.0001, likelihood‐ratio test: *p* < 0.0001, *r* ^2^ = 0.34, sd of interbird variation = 0.17). Latency to begin the task was generally short (average = 9 s ± 2.8 s.e. for completed trials, sd of interbird variation = 0.001) and did not differ significantly based on air temperature (*t* < |0.75| and *p* > 0.47 for both higher temperatures), although during one incomplete trial at 40°C, the bird performed heat dissipation behaviors for over four minutes before beginning the task.

**FIGURE 4:**
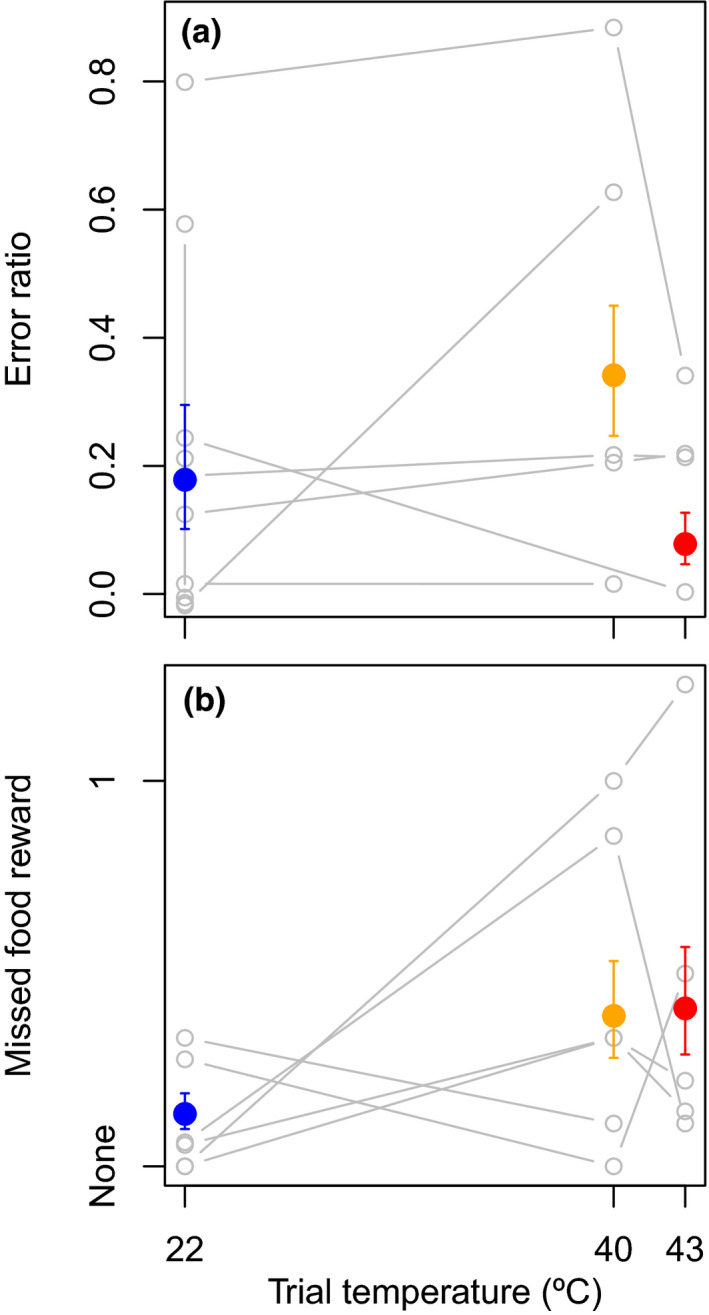
Zebra finches showed evidence of cognitive decline at high air temperatures in one of two aspects of a color association task. Finches maintained high accuracy of color association at higher temperatures (*p* > 0.5 at both 40 and 43°C) (a), though they were more likely to miss food rewards (*p* < 0.05 at both 40 and 43°C) (b). Filled dots and error bars represent model‐based predictions and standard errors; gray lines connect repeated measurements of performance for individuals (*n* = 6)

Birds exhibited declines in one of two cognitive aspects of the color association task at a higher air temperature. Accuracy of the color association task was not related to temperature (Figure 4a, *z* < |1.9| and *p* > 0.05 for both higher temperatures, sd of interbird variation = 1.4). In contrast, birds were significantly more likely to miss food rewards at 40°C (Figure 4b, *η* = −1.15 ± 0.37 s.e., *z* = −3.1, *p* < 0.018) and 43°C (*η* = −1.21 ± 0.39 s.e., *z* = −3.1, *p* < 0.003, likelihood‐ratio test: *p* < 0.002, sd of interbird variation = 0.5). Trial number did not influence performance in any of the above measurements (all *t* < |0.81|, *p* > 0.3), indicating that birds did not improve or decline in performance in successive trials.

### 3.3 Detour‐reaching task

**FIGURE 5:**
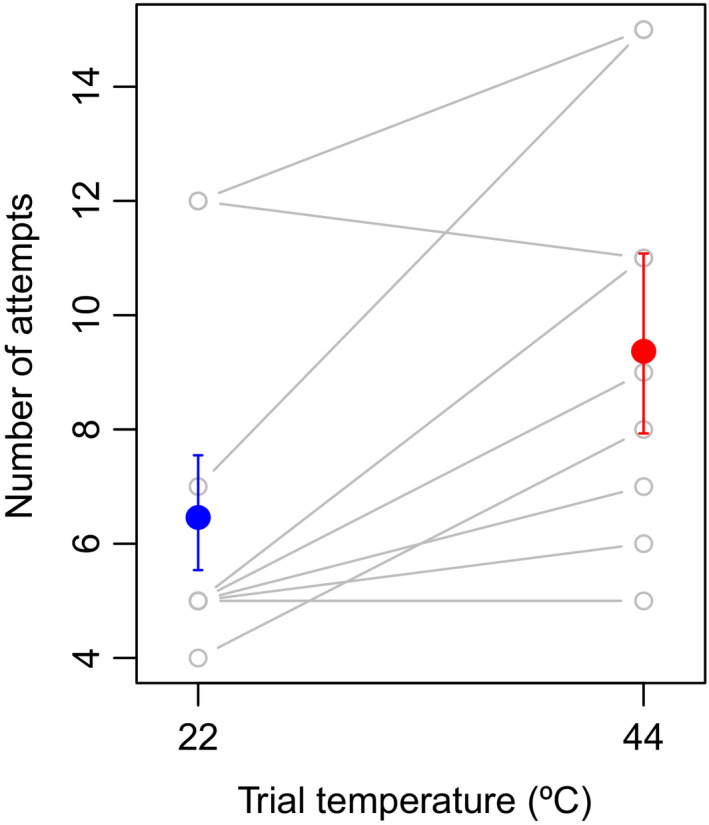
Zebra finches required more trials to complete a detour‐reaching task at a higher air temperature (*p* = 0.03). Filled dots and error bars represent model‐based predictions and standard errors; gray lines connect repeated measurements of performance for individuals (*n* = 9)

Cognitive performance of the detour‐reaching task declined at the high air temperature. Birds required more trials to complete the task at the higher air temperature (Figure 5; *η* = 0.37 (i.e., 2.35 trials) ± 0.17 s.e., *z* = 2.22, *p* < 0.03, *r* ^2^ = 0.26, likelihood‐ratio test: *p* < 0.03, sd of interbird variation = 0.25), indicating lower cognitive performance at the higher temperature. Trial number did not influence performance (*z* = −0.25, *p* = 0.80), indicating that birds did not perform better in later trials as a result of learning in previous trials.

### 3.4 Air temperature in native range

**FIGURE 6:**
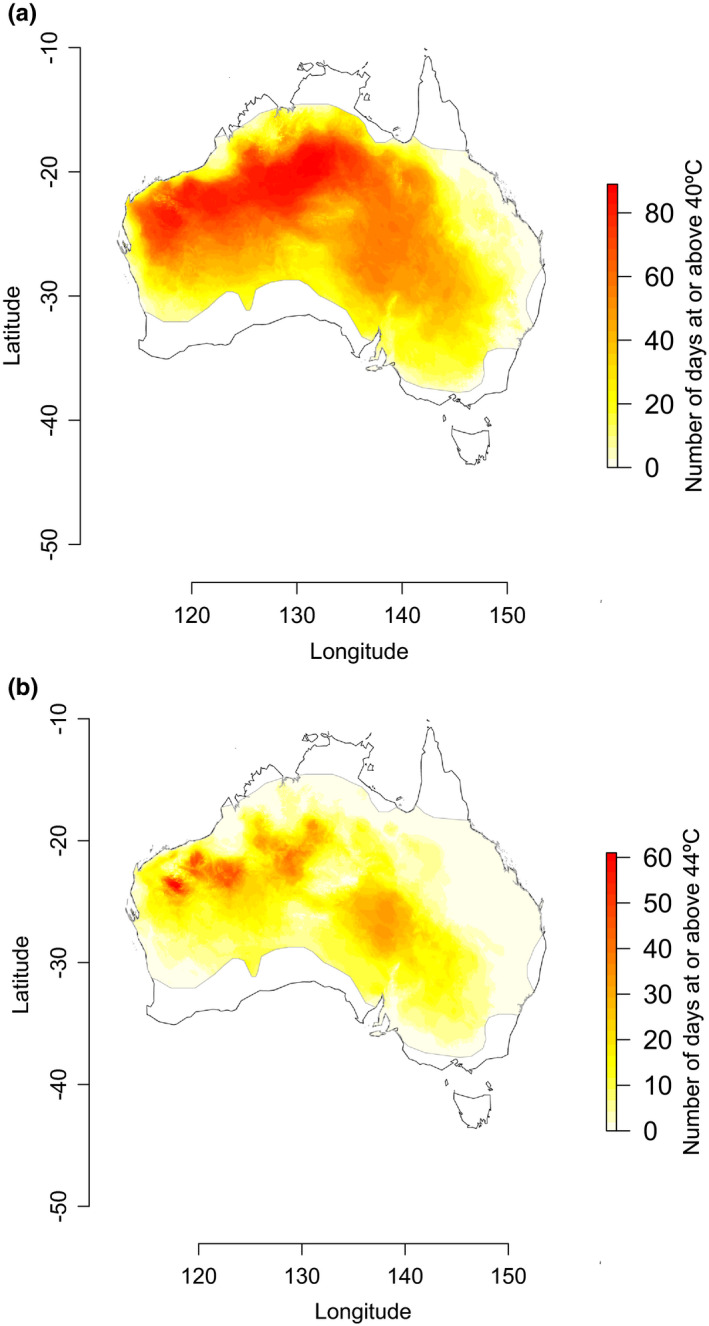
Geographical areas within the zebra finch range (*Taeniopygia guttata castanotis*) during austral summer 2018–2019 in which air temperatures caused (a) motor decline (40°C) and (b) cognitive decline (44°C) in our captive studies. Data taken from the austral summer months of December 2018 to February 2019. Presented as a heat map with the maximum number of days that reached or exceeded that temperature in red and zero days in white. Zebra finch range is outlined in gray

Free‐living zebra finches in Australia experienced air temperatures that caused motor and cognitive declines in our captive birds. During the austral summer months of December 2018–February 2019 temperatures exceeded temperatures causing motor decline (40°C) on up to 89 days (99% of the austral summer days) and exceeded temperatures causing cognitive decline (44°C) on up to 61 days (68% of the austral summer days) (Figure 6). During this time period, zebra finches experienced at least one day at or above 40°C in 45% of their range and 44°C in 34% of their range.

## 4 DISCUSSION

Our findings support the hypothesis that exposure to high air temperatures limits behavioral performance in an endotherm. The behavioral output of animals is composed of motor patterns (e.g., food manipulation) and cognitive processing (e.g., recall of foraging sites), and our findings indicate that both are affected by exposure to high temperatures. At higher air temperatures, when this species is known to experience hyperthermia (Calder, 1964), the birds exhibited heat dissipation behaviors and took longer to complete the color association task, demonstrating reduced motor performance. Birds also required more trials to retrieve the food reward in the detour‐reaching task and missed food rewards during the color association task at higher temperatures, indicating lower cognitive performance on these tasks. Taken together, our findings indicate that high air temperatures can reduce motor and cognitive performance in zebra finches.

Our study shows that reductions in cognitive performance resulted from changes in the thermal environment. Other factors known to influence cognitive performance include interindividual variability in personality (e.g., risk taking), motor ability (e.g., dexterity), motivation (e.g., hunger), and perceptual ability (e.g., visual acuity) (Morand‐Ferron et al., 2016). Our repeated measures design limited the potentially confounding factors of personality, motor ability, and perceptual ability among individuals. Of course, variation in motor ability and even perceptual ability within an individual at different air temperatures is likely part of the explanation for reduced performance. The remaining factor that could explain variability in performance among individuals is motivation. However, we assessed motivation to feed immediately after each assay, and all individuals showed motivation.

We found evidence that exposure to high air temperatures caused motor performance declines through two primary mechanisms. First, birds altered their time budgeted to specific behaviors at high temperatures: birds stopped performing the color association tasks to perform heat dissipation behaviors. This time–budget trade‐off led to slower completion of the color association trials and is consistent with field observations of reduced foraging efficiency while birds perform heat dissipation behaviors (du Plessis et al., 2012). Second, motor speed declined at high air temperatures: birds processed seeds more slowly. Because birds did not actively pant while processing individual seeds, we can be confident that longer seed processing times were a result of slower muscle movement rather than a time–budget trade‐off. Slowed muscle movement could have been intended to reduce heat generation or have resulted from muscle function decline. Indeed, exposure to sublethal air temperatures has been shown to alter muscle physiology in birds (Jimenez & Williams, 2014), which could reduce muscle performance, though this relationship has not been established.

Exposure to high air temperatures also reduced cognitive performance, but not for all cognitive abilities. We found strong effects of temperature on inhibitory behavior. Birds had to inhibit the unproductive behavior of pecking on a clear barrier between themselves and the food reward in order to reach around the tube to acquire the food. The same individuals that could solve this task quickly at a lower air temperature were significantly slower to do so at higher temperatures. In contrast, heat did not have as strong of an effect on discrimination behaviors, as assayed in the color association task. Birds missed food rewards but did not make more mistakes in selecting the correct lid color during color association. Therefore, high air temperatures may have different effects on different cognitive processes. This could be because thermal stress has differential effects on different regions of the brain (Sharma & Hoopes, 2003), warranting further investigation. Further work is also needed to assay additional cognitive processes important to endotherms, such as spatial memory.

The cognitive assays used in our experiments should map onto functional behaviors in the wild. Our color association task measured associative learning. In bumblebees, *Bombus terrestris dalmatians* associative learning speed is correlated with foraging rate (Raine & Chittka, 2008). Our detour‐reaching task measured inhibitory behaviors, which are predictive of problem‐solving skills (Hauser, 1999) and have been correlated with offspring fledged and mating success in wild birds (Cauchard et al., 2013; Keagy et al., 2009; Morand‐Ferron et al., 2016).

The interindividual variation in heat dissipation behaviors and performance declines suggests variability in heat tolerance, which could lead to differential fitness in the wild. Specifically, two of six individuals did not display heat dissipation behaviors at 40°C, whereas all individuals performed these behaviors at 43 and 44°C. Similarly, there was greater variability in aspects of cognitive and motor performance at the higher air temperatures. This is consistent with results from van de Ven et al. (2019), who found that male hornbills (*Tockus leucomelas*) had more variable foraging efficiency in the hottest of the microclimates studied. Luther and Danner (2016) found that male song sparrows with larger bills (which function as heat dissipators) sang more in hot temperatures. Other field studies of birds have not reported interindividual variation in behavioral responses to high air temperatures (Carroll et al., 2015; Funghi et al., 2019; Goldstein, 1984; du Plessis et al., 2012; Wiley & Ridley, 2016). We hypothesize that interindividual variation in behavioral responses to temperature is easier to observe in captive studies, which allow detailed measurement of complex behaviors.

Our study revealed an order to declines in behavioral components at high air temperatures. First, motor and cognitive performance began to decline measurably at different temperatures. Motor decline was evident at 40°C, whereas cognitive decline occurred at 43 and 44°C. Second, motor performance declined sharply between 40 and 43°C. In birds, such temperature response curves are known for some physiological processes (Gerson et al., 2014; McKechnie, Smit, et al., 2016; McKechnie, Whitfield, et al., 2016) and threshold temperatures have been identified for some behaviors (Cunningham, Kruger, et al., 2013), but patterns for cognitive and specific motor processes are poorly known in endotherms. We predict that other endotherms would show similar patterns of cognitive and motor decline, but those specific temperatures at which declines occur vary depending on several factors inherent to the species and its environment.

Our study tested how birds respond to relatively brief exposures to high air temperatures. It is possible that exposure to recurring heat waves or consistently high air temperatures causes acclimatization to, and higher behavioral performance at, high temperatures. For example, acclimation to high temperatures allows some birds to adjust physiological traits such as evaporative cooling (McKechnie & Wolf, 2004). The effects of acclimatization on behavioral performance are poorly understood and deserve study. On the other hand, continuous exposure to high temperatures (e.g., several hours or days) can lead to dehydration and hyperthermia in zebra finches (Calder, 1964), which we hypothesize could lead to even greater declines in behavioral performance than observed in our study. Our work provides a first step to building behavioral response curves at high temperatures and provides a framework for follow‐on studies to address these additional factors.

We assumed that air temperatures in our climate chambers and those used for estimating temperature in the native range represent conditions that zebra finches experience in the wild. Similar to Conradie et al. (2020), we made these assumptions based on a scenario of birds foraging in the shade in a microclimate with air temperature that matches air temperature of the site generally. In the wild, zebra finches likely experience microclimates that match our conditions, as well as those that are cooler and warmer. For example, wind can have a cooling effect, whereas radiation from sunlight and from the ground and vegetation can substantially increase heat gain. Further studies may explore the potential influences of cooler and hotter microclimates on behavioral performance.

Declines in cognitive and motor performance could help predict when and where high air or environmental temperatures will influence population persistence and fitness. McKechnie et al. (2012) described heat‐related mortality in Australian birds and cautioned about greater threats to survival and reproduction in the future. They urged colleagues to create predictive models and provided a conceptual framework for doing so. Our results suggest that zebra finches experienced declines in cognitive and motor performance in portions of their native range in 2018–2019, and paired with future climate scenarios, could provide predictions of behavioral performance declines and mortality in the future. Our results might also help refine existing predictive models that are based on physiology and behavior.

Predictive models of avian mortality in response to heatwaves now exist for Australia (Conradie et al., 2020), the US southwest (Albright et al., 2017), and the Kalahari Desert (Conradie et al., 2019). For Australia and the US southwest, Conradie et al. (2020) and (Albright et al., 2017) used physiological measurements to predict population declines from dehydration and hyperthermia for several species by the end of the century. In Australia, by year 2,100, zebra finches will experience heat waves capable of causing lethal dehydration for over 20 days per year in over 50% of their Australian range and up to 100 days per year in some places (Conradie et al., 2020). In addition, Conradie et al. (2020) provide evidence that zebra finch populations are already declining in the hottest locations and hypothesize that small birds may experience even higher mortality by seeking water in exposed places, which could increase risk of hyperthermia. It is possible that the cognitive and motor performance declines we described in our study exacerbate the threats of dehydration and hyperthermia for birds in the wild by reducing their ability to optimally find water and cooler microhabitats.

For the Kalahari Desert, Conradie et al. (2019) used both physiological and behavioral data to predict that much of the avian biodiversity of that region will be extinct by 2,100 as a result of high air temperatures. Conradie et al. (2019) show that mortality from dehydration and hyperthermia will remain low for birds in shaded locations, and that population declines will result from extended periods of body mass loss, lower nestling growth rates, or breeding failure. Our data support a model of less efficient foraging, which could lead to mass loss and lower nestling mass at high temperatures (Cunningham, Kruger, et al., 2013; van de Ven et al., 2019).

Incorporating cognitive information into predictive models might offer refinements. For example, because cognitive declines occur at higher air temperatures, models of foraging loss might describe increased losses at the highest temperatures. In addition, cognitive and motor decline as described in this study, along with interindividual variation in performance, could provide the basis for predictive models of mate choice (Coomes et al., 2019) and other aspects of breeding biology, which could influence relative fitness and lead to trait evolution.

## CONFLICT OF INTEREST

None declared.

## AUTHOR CONTRIBUTIONS

**Raymond M. Danner:** Conceptualization (equal); data curation (equal); formal analysis (equal); funding acquisition (equal); investigation (equal); methodology (equal); project administration (equal); resources (equal); software (equal); supervision (equal); validation (equal); visualization (equal); writing – original draft (equal); writing – review and editing (equal). **Casey M. Coomes:** Conceptualization (equal); data curation (equal); formal analysis (equal); funding acquisition (equal); investigation (equal); methodology (equal); project administration (equal); resources (equal); software (equal); supervision (equal); validation (equal); visualization (equal); writing – original draft (equal); writing – review and editing (equal). **Elizabeth P. Derryberry:** Conceptualization (equal); data curation (equal); formal analysis (equal); funding acquisition (equal); investigation (equal); methodology (equal); project administration (equal); resources (equal); software (equal); supervision (equal); validation (equal); visualization (equal); writing – original draft (equal); writing – review and editing (equal).

## Data Availability

We provide all R scripts and data on Zenodo (https://zenodo.org/record/4437461). Climate and zebra finch range data are available online and from Birdlife International as described in the Methods section.
